# Characterizing the complete mitogenome of *Heterosentis holospinus* Amin, Heckman & Ha, 2011 (Palaeacanthocephala: Echinorhynchida: Arhythmacanthidae) and its mitochondrial phylogeny

**DOI:** 10.1080/23802359.2025.2528571

**Published:** 2025-07-17

**Authors:** Feiming Chen, Hao Wu, Min Xie, Zhenzhen Xiong, Jia Cai, Yu Huang, Xiao Jin, Can Yang, Jinwei Gao, Rui Song

**Affiliations:** a Fisheries College, Guangdong Provincial Key Laboratory of Aquatic Animal Disease Control and Healthy Culture, Guangdong Ocean University, Zhanjiang, Guangdong, China; b Hunan Fisheries Research Institute and Aquatic Products Seed Stock Station, Changsha, Hunan, China

**Keywords:** Acanthocephala, *Heterosentis holospinus*, Mitochondrial genome, Phylogenetic analysis

## Abstract

*Heterosentis holospinus* Amin, Heckman & Ha, 2011 is a parasitic acanthocephalan, with *Cynoglossus bilineatus* Lacepède, 1802 identified as a new definitive host. We sequenced and annotated its complete mitochondrial genome, which spans 16,560 bp and contains 36 genes, including 22 tRNAs, 12 PCGs and 2 rRNAs. Phylogenetic analysis supports the monophyly of *Heterosentis* and its current taxonomic status. This study enriches essential molecular data that contribute to a clearer phylogenetic framework for Acanthocephala and highlight the utility of mitochondrial genomes in resolving taxonomic relationships at both the genus and family levels.

## 1. Introduction

Acanthocephala, an obligate endoparasitic phylum, comprises three classes: Archiacanthocephala, Palaeacanthocephala, and Eoacanthocephala (Li et al. 2024). They utilize arthropods as intermediate hosts and vertebrates as definitive hosts (Herlyn 2021; Vogel and Taraschewski 2023). Adult parasites attach to host intestinal walls *via* a retractable, spiny proboscis, absorbing nutrients directly from intestinal contents.

*Heterosentis* species infect both freshwater and marine fish across Asia (Gao et al. 2023). *Heterosentis holospinus* Amin, Heckman & Ha, 2011 was originally described from *Plotosus lineatus* Thunberg, 1787 and *Equulites equulus* Forsskål, 1775 (Amin et al. 2011, 2019). This study reports its occurrence in *Cynoglossus bilineatus* Lacepède, 1802, extending its known host range. At least 15 *Heterosentis* species have been described, yet only the mitochondrial genome of *Heterosentis pseudobagri* Wang & Zhang, 1987; Pichelin & Cribb, 1999 is available.

To address this gap and improve phylogenetic resolution, we sequenced and annotated the complete mitochondrial genome of *H. holospinus.* Based on these data, we conducted a phylogenetic analysis involving 35 previously released acanthocephalan mitochondrial genomes to clarify taxonomy and evolutionary relationships.

## 2. Materials and methods

### 2.1. Sample collection, identification and DNA extraction

**Figure 1.**
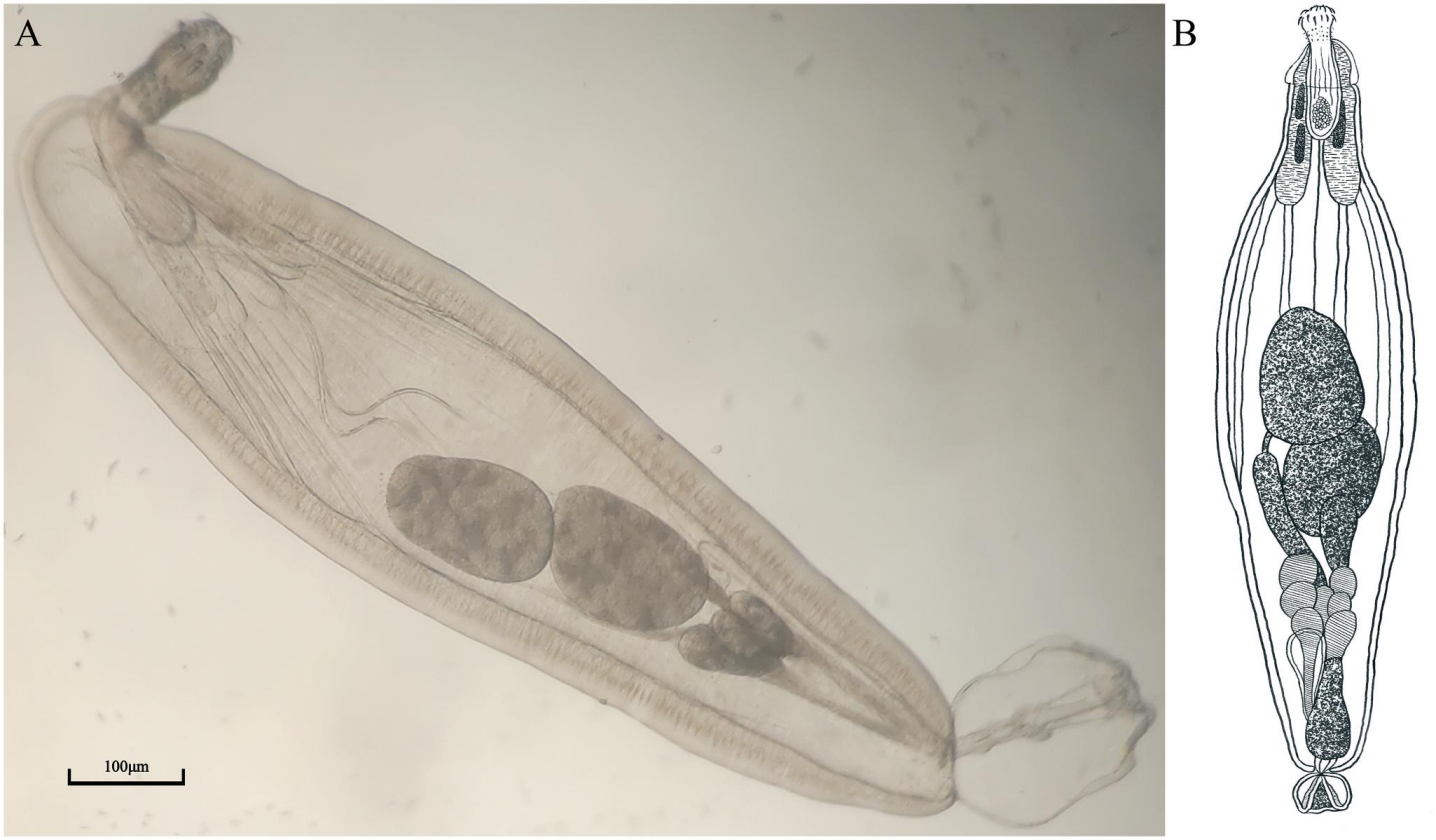
Morphology of the *Heterosentis holospinus*. Photo (A) and sketche (B) were taken and drawn by FMC.

Specimens of *H. holospinus* (Figure 1) were collected from the intestines of refrigerated *C. bilineatus* at a wharf in Zhanjiang, Guangdong Province, China (21°18′50.72″ N, 110°41′81.97″ E). Samples were preserved in absolute ethanol at 4 °C. A specimen was deposited at the Hunan Fisheries Research Institute and Aquatic Products Seed Stock Station (Deposit No. TA20231212; contact person: Jinwei Gao, gaojinwei163@163.com).

Morphological identification followed Amin et al. (2019), confirming key characteristics including a short proboscis: a globular anterior bearing alternating hooks, and a cylindrical posterior with 3-4 rootless hooks per longitudinal row. The double-walled proboscis receptacle is twice the proboscis length. Two elongate lemnisci, each twice the receptacle length, contain one and two giant nuclei, respectively. The body is fusiform, with a spineless anterior trunk cone and a densely spinose remaining trunk. Six cement glands form three longitudinal pairs.

Total genomic DNA was extracted using the TIANamp Micro DNA Kit (Tiangen Biotech, Beijing, China). Specific primers for amplifying the complete mitochondrial genome were designed based on conserved regions identified through alignment with published mitogenomes of closely related acanthocephalan species *H. pseudobagri*. Primers were designed using Primer Premier 5.0 and validated *via* BLASTn against *C. bilineatus* genome to exclude nonspecific binding (Table S1). Long-range PCR with these primers generated overlapping fragments spanning the complete mitogenome. PCR products were resolved on 1.5% agarose gels, and target bands were purified with the AidQuick Gel Extraction Kit (Aidlab Biotech, China). Purified PCR products were sequenced by primer walking strategy at Sangon Biotech (Shanghai) Co., Ltd.. The initial PCR primers served as sequencing primers. Additional internal primers were designed iteratively from new reads to ensure ≥100 bp overlaps. To verify repetitive regions, the corresponding purified PCR products were additionally sequenced with nanopore sequencing technology. Library preparation followed Tyson et al. (2018), and was run on a Qitan Tech QPursue-6k platform at Aoke Biotechnology (Wuhan, China).

### 2.2. Mitogenome assembly and annotation

The mitochondrial genome was assembled with DNASTAR v7.1 (Burland 2000). Transfer RNA genes (tRNAs) were annotated using ARWEN v1.2 (Laslett and Canbäck 2008) and MITOZ v3.4.2 (Meng et al. 2019), with additional verification by comparison to previously published acanthocephalan mitochondrial genomes. A circular map of the mitochondrial genome was generated using Proksee (Grant et al. 2023). Codon usage and relative synonymous codon usage (RSCU) were analyzed using PhyloSuite v1.2.3 (Zhang et al. 2020; Xiang et al. 2023).

### 2.3. Phylogenetic analysis

Given the limited taxonomic representation of mitochondrial genomes within Acanthocephala, all available and reasonably complete mitochondrial genomes of acanthocephalans published till 8 November 2024 were included to establish a comprehensive and informative phylogenetic framework (Table 1). A phylogenetic tree was constructed using 12 protein-coding genes (PCGs) from 36 acanthocephalan species, with *Rotaria rotatoria* Pallas, 1766 and *Philodina citrina* Ehrenberg, 1832 as outgroups. Sequences were extracted using PhyloSuite, aligned with MAFFT v7.471 (Katoh and Standley 2013), and poorly aligned regions were trimmed using TrimAI v1.2 (Capella-Gutiérrez et al. 2009). Saturation tests of the sequences were conducted before concatenating using PhyloSuite. The optimal substitution model (mtInv + F+R5) was selected using ModelFinder v2.2.0 (Kalyaanamoorthy et al. 2017). Maximum likelihood analysis was conducted in IQ-TREE (Nguyen et al. 2015) with 1000 ultrafast bootstraps. The resulting tree was visualized using iTOL (Zhou et al. 2023).

**Table 1.** Detailed information of the representatives of Acanthocephala included in the present phylogeny.

Phylum/class	Order	Family	Species	Accession	Length	AT%	References
Out-group							
Rotifera							
Eurotatoria	Bdelloidea	Philodinidae	*Rotaria rotatoria*	GQ304898	15,319	73.2	Min and Park (2009)
			*Philodina citrina*	FR856884	14,003	77.7	Weber et al. (2013)
In-group							
Acanthocephala							
Archiacanthocephala	Moniliformida	Moniliformidae	*Moniliformis tupaia*	OK415026	14,066	66.2	Dai et al. (2022)
			*Moniliformis sp.*	OP413683	14,150	63.7	Unpublished
	Oligacanthorhynchida	Oligacanthorhynchidae	*Macracanthorhynchus hirudinaceus*	FR856886	14,282	65.2	Weber et al. (2013)
			*Oncicola luehei*	JN710452	14,281	60.2	Gazi et al. (2012)
Eoacanthocephala	Gyracanthocephala	Quadrigyridae	*Acanthogyrus cheni*	KX108947	13,695	65.3	Song et al. (2016)
			*Acanthogyrus bilaspurensis*	MT476589	13,360	59.3	Muhammad et al. (2023)
			*Pallisentis celatus*	JQ943583	13,855	61.5	Pan and Nie (2013)
	Neoechinorhynchida	Neoechinorhynchidae	*Neoechinorhynchus violentum*	KC415004	13,393	59.4	Pan and Jiang (2018)
		Tenuisentidae	*Paratenuisentis ambiguus*	FR856885	13,574	66.9	Weber et al. (2013)
	Polyacanthorhynchida	Polyacanthorhynchidae	*Polyacanthorhynchus caballeroi*	KT592358	13,956	56.3	Gazi et al. (2016)
Palaeacanthocephala	Echinorhynchida	Echinorhynchidae	*Echinorhynchus truttae*	FR856883	13,659	63.1	Weber et al. (2013)
		Arhythmacanthidae	*Heterosentis pseudobagri*	OP278658	13,742	62.5	Gao et al. (2023)
			***Heterosentis holospinus***	**PQ675784**	**16,560**	**61.5**	**This study**
		Cavisomidae	*Cavisoma magnum*	MN562586	13,594	63.0	Muhammad, Li, et al. (2020)
		Leptorhynchoididae	*Brentisentis yangtzensis*	MK651258	13,864	68.3	Song et al. (2019)
		Pomphorhynchidae	*Pomphorhynchus bulbocolli*	JQ824371	13,915	59.9	Unpublished
			*Pomphorhynchus laevis*	JQ809446	13,889	57.1	Unpublished
			*Pomphorhynchus rocci*	JQ824373	13,845	60.7	Unpublished
			*Pomphorhynchus tereticollis*	JQ809451	13,965	56.9	Unpublished
			*Longicollum sp.*	OR215045	14,632	55.8	Unpublished
		Pseudoacanthocephalidae	*Pseudoacanthocephalus sp.*	OQ588705	14,883	61.5	Unpublished
			*Pseudoacanthocephalus bufonis*	MZ958236	14,056	58.4	Zhao et al. (2023)
		Rhadinorhynchidae	*Leptorhynchoides thecatus*	AY562383	13,888	71.4	Steinauer et al. (2005)
			*Micracanthorhynchina dakusuiensis*	OP131911	16,309	56.8	Gao et al. (2022)
	Polymorphida	Centrorhynchidae	*Centrorhynchus clitorideus*	MT113355	15,884	55.5	Muhammad, Khan, et al. (2020)
			*Centrorhynchus milvus*	MK922344	14,314	54.5	Muhammad et al. (2019b)
			*Centrorhynchus aluconis*	KT592357	15,144	55.6	Gazi et al. (2016)
			*Sphaerirostris lanceoides*	MT476588	13,478	58.0	Muhammad, Ahmad, et al. (2020)
			*Sphaerirostris picae*	MK471355	15,170	58.1	Muhammad et al. (2019a)
			*Southwellina hispida*	KJ869251	14,742	63.9	Gazi et al. (2015)
			*Bolbosoma nipponicum*	OR468096	14,296	60.9	Li et al. (2024)
			*Bolbosoma balaenae*	MZ357084	14,301	62.6	García-Gallego et al. (2023)
			*Bolbosoma capitatum*	MZ357085	14,319	63.9	García-Gallego et al. (2023)
			*Bolbosoma vasculosum*	MZ357087	14,313	63.9	García-Gallego et al. (2023)
			*Corynosoma villosum*	OR468095	14,241	60.9	Li et al. (2024)
		Plagiorhynchidae	*Plagiorhynchus transversus*	KT447549	15,477	61.1	Gazi et al. (2016)

## 3. Results

**Figure 2.**
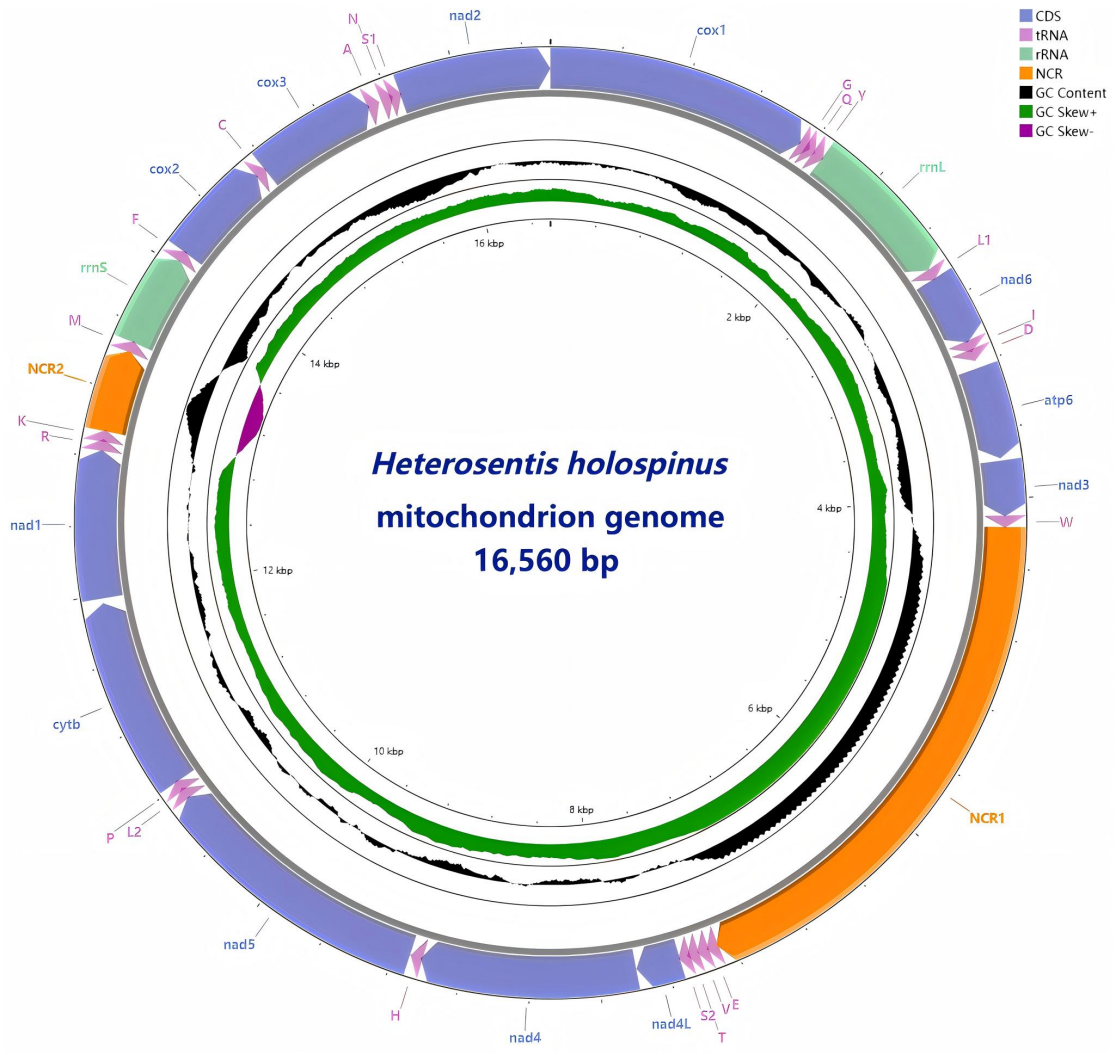
The circular gene maps of the mitochondrial genomes of *H. holospinus*. The innermost and Middle circles depict the GC-skew and GC content, respectively. The outermost circle indicates the arrangements of genes: inner genes from the reverse strand, and outer genes from the forward strand, with PCGs in bluish violet, rRNAs in light green, NCRs light orange, and tRNAs in light purple.

*H. holospinus* is identified based on morphological characteristics and *cox1* sequence analysis (Supplementary Figure S1). Its complete mitochondrial genome is 16,560 bp in length (GenBank accession No. PQ675784). It contains 36 genes, including 22 tRNAs, 12 PCGs, and 2 ribosomal RNA genes (*rrnS* and *rrnL*). Notably, two non-coding regions (NCRs) were identified, and the *atp8* gene is absent. All genes are encoded on the heavy strand (Figure 2). The gene number and arrangement are consistent with the same genus species *H. pseudobagri.* The overall A + T content of 61.5%, with a nucleotide composition of 37.5% T, 24.0% A, 29.3% G, and 9.2% C. The AT skew and GC skew are −0.22 and 0.521, respectively. Among the 12 PCGs, eight PCGs initiate with GTG, three with ATG, and one with ATT. Four PCGs terminate with TAG, two TAA, and six truncated T. Valine (Val) is the most abundant amino acid, and UUA (Leu) is the most frequently used codon. In contrast, glutamine (Gln) is the least abundant amino acid, while CGC is the rarest codon, appearing only once (Supplementary Figure S2).

**Figure 3.**
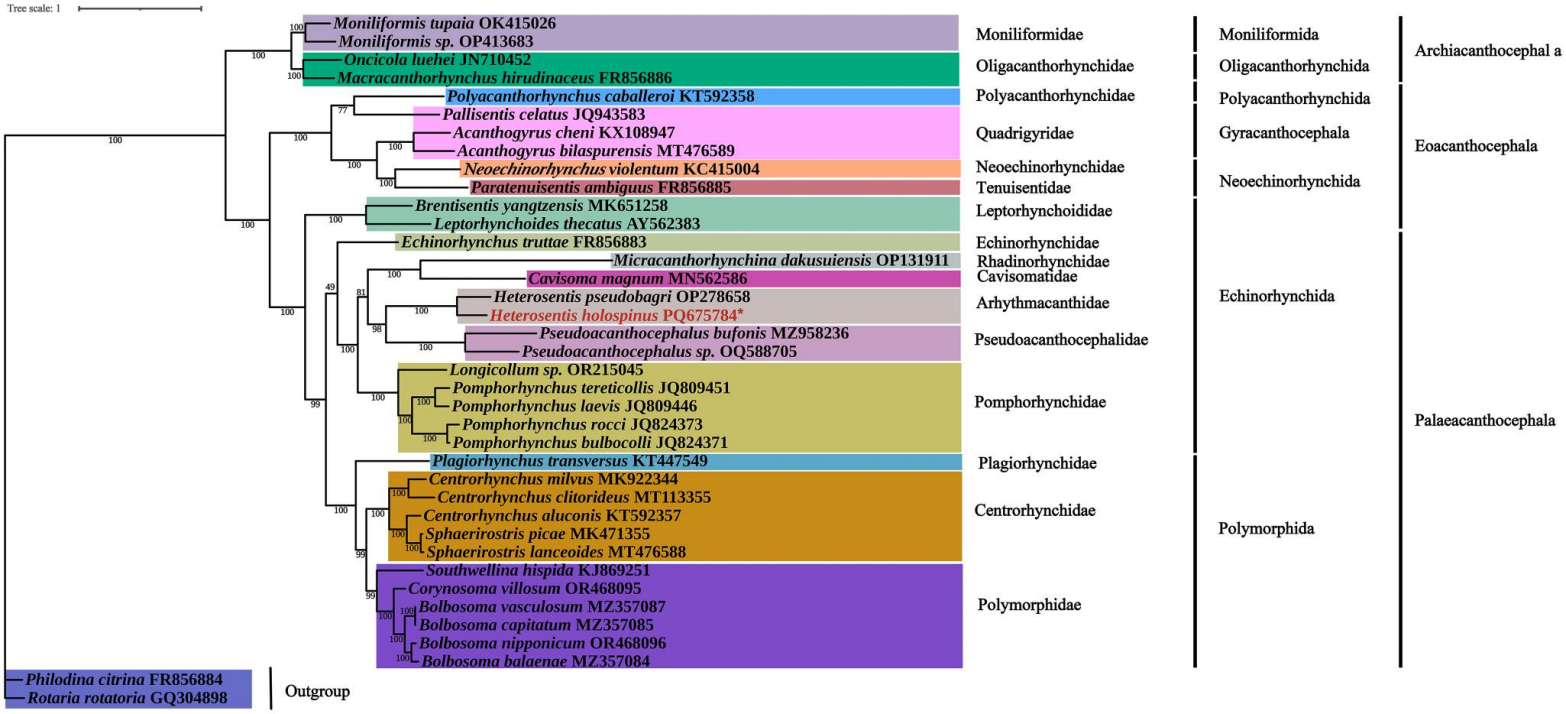
Phylogenetic tree constructed based on 12 PCGs. The phylogenetic tree shows the evolutionary relationships between heterosentis holospinus and 35 other acanthocephalas species, with *Rotaria rotatoria* and *Philodina citrina* as the outgroups.

A regression analysis yielded an *R*^2^ value of 0.737, indicating a moderately strong correlation (Supplementary Figure S3). Phylogenetic analysis revealed that *H. holospinus* clustered with *H. pseudobagri* forming a well-supported sister clade representing the genus *Heterosentis*. This clade was further clustered with members of the genus *Pseudoacanthocephalus*, and sequentially clustered with lineages representing other genera or families within the order Echinorhynchida (Figure 3).

## 4. Discussion and conclusions

In this study, we sequenced and annotated the complete mitochondrial genome of *H. holospinus*, expanding the available molecular dataset for the genus *Heterosentis*. With a length of 16,560 bp, this mitogenome is currently the longest reported among acanthocephalans, primarily due to a lengthy tandem repeat region. Similar tandem repeats have been identified in *Micracanthorhynchina dakusuiensis* Harada, 1938 and *Pallisentis celatus* Van Cleave, 1928, but are absent in the closely related *H. pseudobagri*. The evolutionary significance of these tandem repeats remains unclear, and warrants further comparative studies across more acanthocephalan lineages (Pan and Nie 2013; Gao et al. 2022). *C. bilineatus* was identified as a new definitive host for *H. holospinus*, extending its known host range. This finding underscores the ecological adaptability of *H. holospinus* and may have implications for understanding host-parasite dynamics in both marine and estuarine ecosystems. Phylogenetic analysis supports the monophyly of the genus *Heterosentis*, supporting previous morphological and molecular findings (Amin et al. 2019; Gao et al. 2023). Overall, this study enriches essential molecular data that contribute to a clearer phylogenetic framework for Acanthocephala, and highlight the utility of mitochondrial genomes in resolving taxonomic relationships at both the genus and family levels.

## Supplementary Material

Fig 2 Genome Feature Map.zip

Fig 1 Species Reference Image.zip

Supplementary Table S1 Primers and PCR gel plot.docx

Fig 3 Phylogenetic tree constructed based on 12 PCGs.zip

## Data Availability

The BioProject, BioSample, and SRA accession numbers related to the sample in this study are PRJNA1202589, SAMN45962552, and SRS23636456, respectively. The mitochondrial genome sequence supporting the findings of this study is openly available in the National Center for Biotechnology Information (NCBI) with GenBank accession number PQ675784. Chromatography files have been deposited in ScienceDB (https://doi.org/10.57760/sciencedb.18676).

## References

[CIT0001] Amin OM, Heckmann RA, Van Ha N. 2011. Description of *Heterosentis holospinus n. sp.* (Acanthocephala: arhythmacanthidae) from the striped eel catfish, *Plotosus lineatus*, in Halong bay, Vietnam, with a key to species of *Heterosentis* and reconsideration of the subfamilies of Arhythmacanthidae. Comparat Parasitol. 78(1):29–38. doi:10.1654/4465.1.

[CIT0002] Amin OM, Rodríguez SM, Heckmann RA. 2019. Morphological updates and molecular description of *Heterosentis holospinus* Amin, Heckmann, & Ha, 2011 (Acanthocephala, Arhythmacanthidae) in the Pacific Ocean off Vietnam. Parasite. 26:73. doi:10.1051/parasite/2019072. 31855174 PMC6921964

[CIT0003] Burland TG. 2000. DNASTAR’s Lasergene sequence analysis software. Methods Mol Biol. 132:71–91. doi:10.1385/1-59259-192-2:71. 10547832

[CIT0004] Capella-Gutiérrez S, Silla-Martínez JM, Gabaldón T. 2009. trimAI: a tool for automated alignment trimming in large-scale phylogenetic analyses. Bioinformatics. 25(15):1972–1973. doi:10.1093/bioinformatics/btp348. 19505945 PMC2712344

[CIT0005] Dai GD, Yan HB, Li L, Zhang LS, Liu ZL, Gao SZ, Ohiolei JA, Wu YD, Guo AM, Fu BQ, et al. 2022. Molecular characterization of a new *Moniliformis sp.* from a Plateau Zokor (*Eospalax fontanierii baileyi*) in China. Front Microbiol. 13:806882. doi:10.3389/fmicb.2022.806882. 35356531 PMC8959414

[CIT0006] Gao JW, Yuan XP, Jakovlić I, Wu H, Xiang CY, Xie M, Song R, Xie ZG, Wu YA, Ou DS. 2023. The mitochondrial genome of *Heterosentis pseudobagri* (Wang & Zhang, 1987) Pichelin & Cribb, 1999 reveals novel aspects of tRNA genes evolution in Acanthocephala. BMC Genomics. 24(1):95. doi:10.1186/s12864-023-09177-9. 36864372 PMC9979467

[CIT0007] Gao JW, Yuan XP, Wu H, Xiang CY, Xie M, Song R, Chen ZY, Wu YA, Ou DS. 2022. Mitochondrial phylogenomics of Acanthocephala: nucleotide alignments produce long-branch attraction artefacts. Parasit Vectors. 15(1):376. doi:10.1186/s13071-022-05488-0. 36261865 PMC9583589

[CIT0008] García-Gallego A, Raga JA, Fraija-Fernández N, Aznar FJ. 2023. Temporal and geographical changes in the intestinal helminth fauna of striped dolphins, *Stenella coeruleoalba*, in the western Mediterranean: a long-term analysis (1982–2016). Front Mar Sci. 10:17. doi:10.3389/fmars.2023.1272353.

[CIT0009] Gazi M, Kim J, García‐Varela M, Park C, Littlewood DTJ, Park JK. 2016. Mitogenomic phylogeny of Acanthocephala reveals novel class relationships. Zool Scr. 45(4):437–454. doi:10.1111/zsc.12160.

[CIT0010] Gazi M, Kim J, Park JK. 2015. The complete mitochondrial genome sequence of *Southwellina hispida* supports monophyly of Palaeacanthocephala (Acanthocephala: polymorphida). Parasitol Int. 64(4):64–68. doi:10.1016/j.parint.2015.01.009. 25656507

[CIT0011] Gazi M, Sultana T, Min GS, Park YC, García-Varela M, Nadler SA, Park JK. 2012. The complete mitochondrial genome sequence of *Oncicola luehei* (Acanthocephala: archiacanthocephala) and its phylogenetic position within Syndermata. Parasitol Int. 61(2):307–316. doi:10.1016/j.parint.2011.12.001. 22198415

[CIT0012] Grant JR, Enns E, Marinier E, Mandal A, Herman EK, Chen CY, Graham M, Van Domselaar G, Stothard P. 2023. Proksee: in-depth characterization and visualization of bacterial genomes. Nucleic Acids Res. 51(W1):W484–W492. doi:10.1093/nar/gkad326. 37140037 PMC10320063

[CIT0013] Herlyn H. 2021. Thorny-headed worms (Acanthocephala): jaw-less members of jaw-bearing worms that parasitize jawed arthropods and jawed vertebrates. Evol Fossil Rec Parasitism. 49:273–313.

[CIT0014] Kalyaanamoorthy S, Minh BQ, Wong TKF, von Haeseler A, Jermiin LS. 2017. ModelFinder: fast model selection for accurate phylogenetic estimates. Nat Methods. 14(6):587–589. doi:10.1038/nmeth.4285. 28481363 PMC5453245

[CIT0015] Katoh K, Standley DM. 2013. MAFFT multiple sequence alignment software version 7: improvements in performance and usability. Mol Biol Evol. 30(4):772–780. doi:10.1093/molbev/mst010. 23329690 PMC3603318

[CIT0016] Laslett D, Canbäck B. 2008. ARWEN: a program to detect tRNA genes in metazoan mitochondrial nucleotide sequences. Bioinformatics. 24(2):172–175. doi:10.1093/bioinformatics/btm573. 18033792

[CIT0017] Li DX, Yang RJ, Chen HX, Kuzmina TA, Spraker TR, Li L. 2024. Characterization of the complete mitochondrial genomes of the zoonotic parasites *Bolbosoma nipponicum* and *Corynosoma villosum* (Acanthocephala: Polymorphida) and the molecular phylogeny of the order Polymorphida. Parasitology. 151(1):45–57. doi:10.1017/S0031182023001099. 37955106 PMC10941042

[CIT0018] Meng GL, Li YY, Yang CT, Liu SL. 2019. MitoZ: a toolkit for animal mitochondrial genome assembly, annotation and visualization. Nucleic Acids Res. 47(11):e63–e63. doi:10.1093/nar/gkz173. 30864657 PMC6582343

[CIT0019] Min GS, Park JK. 2009. Eurotatorian paraphyly: revisiting phylogenetic relationships based on the complete mitochondrial genome sequence of *Rotaria rotatoria* (Bdelloidea: Rotifera: Syndermata). BMC Genomics. 10(1):533. doi:10.1186/1471-2164-10-533. 19919696 PMC2784805

[CIT0020] Muhammad N, Ahmad MS, Li L, Zhao Q, Ullah H, Zhu X-Q, Ma J, Suleman. 2020. Mitochondrial DNA dataset suggest that the genus *Sphaerirostris* Golvan, 1956 is a synonym of the genus *Centrorhynchus* Lühe, 1911. Parasitology, 147(10): 1149–1157. doi:10.1017/S0031182020000906. 32487273 PMC10317752

[CIT0021] Muhammad N, Khan MS, Li L, Zhao Q, Ullah H, Zhu X-Q, Ma J, Suleman. 2020c. Characterization of the complete mitogenome of *Centrorhynchus clitorideus* (Meyer, 1931) (Palaeacanthocephala: Centrorhynchidae), the largest mitochondrial genome in Acanthocephala, and its phylogenetic implications. Mol Biochem Parasitol, 237: 111274. doi:10.1016/j.molbiopara.2020.111274. 32243910

[CIT0022] Muhammad N, Li D-X, Ru S-S, Saood D, Alvi MA, Li L, Suleman. 2023. Characterization of the complete mitochondrial genome of *Acanthogyrus (Acanthosentis) bilaspurensis* Chowhan, Gupta & Khera, 1987 (Eoacanthocephala: Quadrigyridae), the smallest mitochondrial genome in Acanthocephala, and its phylogenetic implications. J Helminthol, 97: e87. doi:10.1017/S0022149X23000561. 37969070

[CIT0023] Muhammad N, Li L, Zhao Q, Bannai MA, Mohammad ET, Khan MS, Zhu X-Q, Ma J, Suleman. 2020. Characterization of the complete mitochondrial genome of *Cavisoma magnum* (Southwell, 1927) (Acanthocephala: Palaeacanthocephala), first representative of the family Cavisomidae, and its phylogenetic implications. Infect Genet Evol, 80: 104173. doi:10.1016/j.meegid.2020.104173. 31917357

[CIT0024] Muhammad N, Ma J, Khan MS, Li L, Zhao Q, Ahmad MS, Zhu X-Q, Suleman. 2019a. Characterization of the complete mitochondrial genome of *Sphaerirostris picae* (Rudolphi, 1819) (Acanthocephala: centrorhynchidae), representative of the genus *Sphaerirostris*. Parasitol Res, 118(7): 2213–2221. doi:10.1007/s00436-019-06356-0. 31183599

[CIT0025] Muhammad N, Ma J, Khan MS, Wu S-S, Zhu X-Q, Li L, Suleman. 2019b. Characterization of the complete mitochondrial genome of *Centrorhynchus milvus* (Acanthocephala: polymorphida) and its phylogenetic implications. Infect Genet Evol, 75: 103946. doi:10.1016/j.meegid.2019.103946. 31279002

[CIT0026] Nguyen LT, Schmidt HA, von Haeseler A, Minh BQ. 2015. IQ-TREE: a fast and effective stochastic algorithm for estimating maximum-likelihood phylogenies. Mol Biol Evol. 32(1):268–274. doi:10.1093/molbev/msu300. 25371430 PMC4271533

[CIT0027] Pan TS, Jiang H. 2018. The complete mitochondrial genome of *Hebesoma violentum* (Acanthocephala). Mitochondrial DNA B Resour. 3(2):582–583. doi:10.1080/23802359.2018.1473717. 33474250 PMC7800221

[CIT0028] Pan TS, Nie P. 2013. The complete mitochondrial genome of *Pallisentis celatus* (Acanthocephala) with phylogenetic analysis of acanthocephalans and rotifers. Folia Parasitol (Praha). 60(3):181–191. doi:10.14411/fp.2013.021. 23951925

[CIT0029] Song R, Zhang D, Deng SM, Ding DM, Liao FC, Liu LS. 2016. The complete mitochondrial genome of *Acanthosentis cheni* (Acanthocephala: Quadrigyridae). Mitochondrial DNA B Resour. 1(1):797–798. doi:10.1080/23802359.2016.1197076. 33473631 PMC7799462

[CIT0030] Song R, Zhang D, Gao JW, Cheng XF, Xie M, Li H, Wu YA. 2019. Characterization of the complete mitochondrial genome of *Brentisentis yangtzensis* Yu & Wu, 1989 (Acanthocephala, Illiosentidae). Zookeys. 861:1–14. doi:10.3897/zookeys.861.34809. 31363345 PMC6656981

[CIT0031] Steinauer ML, Nickol BB, Broughton R, Ortí G. 2005. First sequenced mitochondrial genome from the phylum Acanthocephala (*Leptorhynchoides thecatus*) and its phylogenetic position within Metazoa. J Mol Evol. 60(6):706–715. doi:10.1007/s00239-004-0159-8. 15909226

[CIT0032] Tyson JR, O’Neil NJ, Jain M, Olsen HE, Hieter P, Snutch TP. 2018. MinION-based long-read sequencing and assembly extends the Caenorhabditis elegans reference genome. Genome Res. 28(2):266–274. doi:10.1101/gr.221184.117. 29273626 PMC5793790

[CIT0033] Vogel S, Taraschewski H. 2023. Intermediate host patterns of acanthocephalans in the Weser river system: co-invasion *vs* host capture. Parasitology. 150(5):426–433. doi:10.1017/S0031182023000124. 36793230 PMC10089806

[CIT0034] Weber M, Wey-Fabrizius AR, Podsiadlowski L, Witek A, Schill RO, Sugár L, Herlyn H, Hankeln T. 2013. Phylogenetic analyses of endoparasitic Acanthocephala based on mitochondrial genomes suggest secondary loss of sensory organs. Mol Phylogenet Evol. 66(1):182–189. doi:10.1016/j.ympev.2012.09.017. 23044398

[CIT0035] Xiang CY, Gao FL, Jakovlić I, Lei HP, Hu Y, Zhang H, Zou H, Wang GT, Zhang D. 2023. Using PhyloSuite for molecular phylogeny and tree‐based analyses. Imeta. 2(1):e87. doi:10.1002/imt2.87. 38868339 PMC10989932

[CIT0036] Zhang D, Gao FL, Jakovlić I, Zou H, Zhang J, Li WX, Wang GT. 2020. PhyloSuite: an integrated and scalable desktop platform for streamlined molecular sequence data management and evolutionary phylogenetics studies. Mol Ecol Resour. 20(1):348–355. doi:10.1111/1755-0998.13096. 31599058

[CIT0037] Zhou T, Xu KD, Zhao F, Liu WY, Li LZ, Hua ZY, Zhou X. 2023. itol.toolkit accelerates working with iTOL (interactive tree of life) by an automated generation of annotation files. Bioinformatics. 39(6): 1–4. doi:10.1093/bioinformatics/btad339. PMC10243930 37225402

