# Supplementary material for: Characterizing the complete mitogenome of Heterosentis holospinus Amin, Heckman & Ha, 2011 (Palaeacanthocephala: Echinorhynchida: Arhythmacanthidae) and its mitochondrial phylogeny
Source: Mitochondrial DNA B Resour. 2025 Jul 17;10(8):742–7. doi: 10.1080/23802359.2025.2528571 (PMC12272951; doi:10.1080/23802359.2025.2528571)
Supplement: Supplementary Table S1 Primers and PCR gel plot.docx [file TMDN_A_2528571_SM8523.docx]

**Table S1.** Primers used for amplification of the mitochondrial genome of *Heterosentis holospinus*

| Fragment  No. | Gene or  region | Primer  name | Sequence (5’-3’) | Length  (bp) |
| --- | --- | --- | --- | --- |
| F1 | *cox1* | STF1 | GTTTTGGTTTTTTGGGCATC | 623 |
|  |  | STR1 | CTATATAACGACGAGGTATACC |  |
| F2 | *cox1-rrnL* | STF2 | GATGGGTGTTATGTTTTCAATTG | 1200 |
|  |  | STR2 | ATTCTAGGGTCTTTCCGTCT |  |
| F3 | *rrnL* | STF3 | GACTATGCTAAGGTAGCATA | 330 |
|  |  | STR3 | CTCACACCGATCTAAACTCA |  |
| F4 | *rrnL-nad4* | STF4 | ACTCTAGGGATAACAGGGTA | 6079 |
|  |  | STR4 | ACAGCTACAACAATGGTTAG |  |
| F5 | *nad4* | STF5 | CATGGGTGATTACCCAAAG | 244 |
|  |  | STR5 | GGATAATTGATGAATATGC |  |
| F6 | *nad4-nad5* | STF6 | CCATTGTTGTAGCTGTTTGG | 1105 |
|  |  | STR6 | CCTACAACCATACTTAACATAAG |  |
| F7 | *nad5* | STF7 | ATTGGTGGGGTGGGAGAT | 306 |
|  |  | STR7 | GAATGGACTAAAGCAGACAC |  |
| F8 | *nad5-cytb* | STF8 | TAGGTTGGGAAGGTGGAAGT | 1728 |
|  |  | STR8 | GAGTAGTGATGCTTCTACCT |  |
| F9 | *cytb* | STF9 | CTTGGGGTACGTCCTCCCT | 434 |
|  |  | STR9 | ACCACTCAGGTTTAATATG |  |
| F10 | *cytb-rrnS* | STF10 | GTGTTTGTGTATTGGTTAGTAG | 2231 |
|  |  | STR10 | CTAAATACCTGGGTCTCTAATCCAG |  |
| F11 | *rrnS* | STF11 | CAGCGTCAGCGGTTATAC | 408 |
|  |  | STR11 | TGTTACGACTTACACCTT |  |
| F12 | *rrnS-cox2* | STF12 | GAAGAGTGCATAAATGTTTAGAG | 747 |
|  |  | STR12 | CCTAAAACCTCTACACCAACCTG |  |
| F13 | *cox2* | STF13 | GATGTGATTCATTCTTGGGC | 131 |
|  |  | STR13 | CCACATAATTCAGAACACTG |  |
| F14 | *cox2-cox1* | STF14 | GGTGTTAAAGTTGATTGTATTCC | 2780 |
|  |  | STR14 | ACCCAAACACTTCAAACTTACC |  |

PCR gel plot


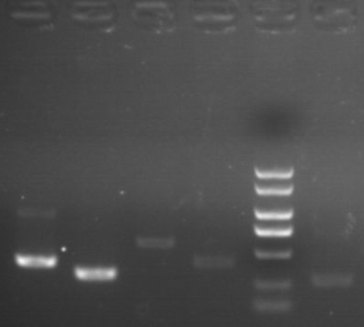


Fig. P1 **Target bands fragments from left to right: STF11R11 (*rrnS*: 408 bp), STF3R3 (*rrnL*: 330 bp), STF1R1 (cox*1*: 408 bp), STF9R9 (*cytb*: 434 bp), STF7R7 (*nad5*: 306 bp).**

**DL2000 Plus DNA Marker: 2000, 1500, 1000, 750, 500, 250, 100 bp.**


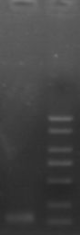


Fig. P2 **Target band fragment of STF13R13 (*cox2*: 131 bp).**

**DL2000 Plus DNA Marker: 2000, 1500, 1000, 750, 500, 250, 100 bp.**


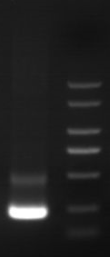


Fig. P3 **Target band fragment of STF5R5 (*nad4*: 244 bp)**

**DL2000 Plus DNA Marker: 2000, 1500, 1000, 750, 500, 250, 100 bp.**


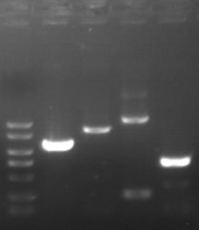


Fig. P4 **Target bands fragment From left to right: STF2R2 (*cox1-rrnL*: 1200 bp), STF8R8 (*nad5-cytb*: 1728 bp), STF10R10 (*cytb-rrnS*: 2231 bp), STF12R12 (*rrnS-cox2*: 747 bp)**

**DL2000 Plus DNA Marker: 2000, 1500, 1000, 750, 500, 250, 100 bp.**


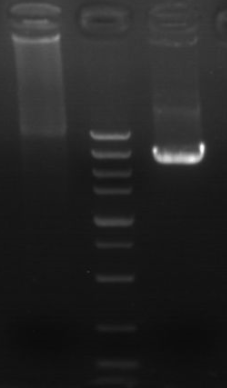


Fig. P5 **Target band fragment of STF14R14 (*cox2-cox1*: 2780 bp)**

**DL5000 DNA Marker: 5000, 3000, 2000, 1500, 1000, 750, 500, 250, 100 bp.**


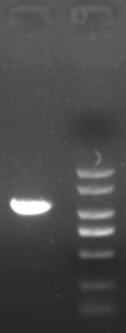


Fig. P6 **Target band fragment of STF6R6 (*nad4-nad5*: 1105 bp)**

**DL2000 Plus DNA Marker: 2000, 1500, 1000, 750, 500, 250, 100 bp.**


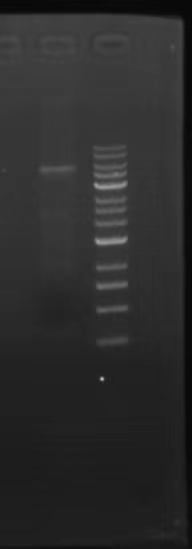


Fig. P7 **Target band fragment of STF4R4 (*rrnL-nad4*: 6079 bp)**

**DL1000 DNA Marker: 10000, 8000, 6000, 5000, 4000, 3000, 2000, 1000 bp.**
